# Decoding the essential interplay between central and peripheral control in adaptive locomotion of amphibious centipedes

**DOI:** 10.1038/s41598-019-53258-3

**Published:** 2019-12-02

**Authors:** Kotaro Yasui, Takeshi Kano, Emily M. Standen, Hitoshi Aonuma, Auke J. Ijspeert, Akio Ishiguro

**Affiliations:** 1 Research Institute of Electrical Communication, Tohoku University, 2-1-1 Katahira, Aoba-Ward, Sendai, 980-8577 Japan; 2 Japan Society for the Promotion of Science (JSPS), 5-3-1 Kojimachi, Chiyoda-Ward, Tokyo 102-0083 Japan; 3 Department of Biology, University of Ottawa, 30 Marie Curie Private, Ottawa, Ontario K1N 6N5 Canada; 4 Research Institute for Electronic Science, Hokkaido University, N12W7, Kita-Ward, Sapporo 060-0812 Japan; 5 Institute of Bioengineering, École Polytechnique Fédérale de Lausanne (EPFL), Lausanne, CH-1015 Switzerland

**Keywords:** Computational models, Dynamical systems

## Abstract

Amphibious animals adapt their body coordination to compensate for changing substrate properties as they transition between terrestrial and aquatic environments. Using behavioural experiments and mathematical modelling of the amphibious centipede *Scolopendra subspinipes mutilans*, we reveal an interplay between descending command (brain), local pattern generation, and sensory feedback that controls the leg and body motion during swimming and walking. The elongated and segmented centipede body exhibits a gradual transition in the locomotor patterns as the animal crosses between land and water. Changing environmental conditions elicit a mechano-sensory feedback mechanism, inducing a gait change at the local segment level. The body segments operating downstream of a severed nerve cord (no descending control) can generate walking with mechano-sensory inputs alone while swimming behaviour is not recovered. Integrating the descending control for swimming initiation with the sensory feedback control for walking in a mathematical model successfully generates the adaptive behaviour of centipede locomotion, capturing the possible mechanism for flexible motor control in animals.

## Introduction

Flexible coordination of body movement in animals enables adaptive locomotion across changing environments or landscapes. In particular, the transitions between terrestrial and aquatic environments require flexibility of motor control since the physical properties of the substrates are significantly different. For example, amphibian salamanders swim in water by propagating axial bending waves with their limbs folded along the body trunk, whereas on land they show a quadrupedal gait with a standing wave of body undulation^1^. Such an adaptive behaviour is achieved by changing the coordination patterns of many degrees of freedom in the body depending on the substrates, and this strategy is widely observed in various amphibious animals, e.g. fish^2–4^, turtles^5^, and insects^6,7^. Decoding the essential control mechanism in amphibious locomotion may lead to understanding the common principles of flexible motor control in animals, and its applications will help enhance the adaptability of robotic locomotion to environmental changes.

The key to tackling this problem is to capture the interplay between descending commands from the brain, local pattern generating circuits (i.e. central pattern generator [CPG]^8^) and sensory feedback. In the long history of neurobiology of locomotion control, the abovementioned three elements are considered to play important roles, and the independent function of each element has been clarified to some extent^8–11^. The interplay between the three, however, mostly remains elusive, as capturing the complexity of interactions require simultaneous measurement of each type of neural activity, which is technically difficult. In fact, very little is known of the inherent neural control mechanisms which generate the adaptive change in body coordination during amphibious locomotion. For salamanders, it was reported that two distinct locomotor patterns of walking and swimming can be switched in response to the magnitude of descending command from the brain^12^, and the possible neural circuit was modelled and validated by physical robot experiments^13^. These studies, though pivotal, did not address how amphibious animals sense environmental changes, and therefore, it is still unclear to what extent local sensory feedback mechanisms are responsible for switching between different locomotor patterns.

**Figure 1:**
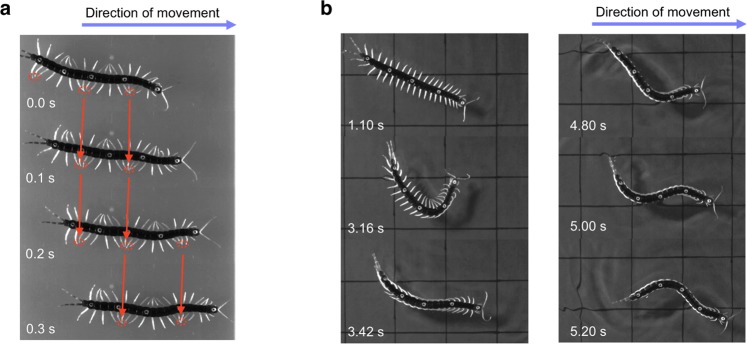
Representative sequences of a centipede walking and swimming. (**a**) Walking on land as observed from the top view. Snapshots were taken every 0.1 s. Red dotted circles indicate dense part of the leg tip positions of the right side. Red arrows denote backward propagation of the wave of leg movement along the body axis. (**b**) Swimming in water. The first three snapshots (from 1.10 s to 3.42 s) show the initiation of swimming after the centipede was submerged in water. The remaining three snapshots (from 4.80 s to 5.20 s) show typical swimming motion with travelling axial bending waves and leg folding.

Herein, we focused on the centipede (*Scolopendra subspinipes mutilans*) as a model animal. Like the salamander, the centipede exhibits completely different body-limb coordination during amphibious locomotion. It swims in water with a travelling body undulation movement, whereas on land, it walks by propagating a wave of leg movement with less body undulation^14^ (Fig. 1). Most importantly, the homogeneous and segmented body structure of the centipede provides a great opportunity to investigate the role of local sensory feedback in switching motor coordination since the elongate, legged body segments of the centipede facilitate the visualization of kinematic changes in the body as the animal crosses between terrestrial and aquatic environments. In addition, lesion experiments that alter the continuity of neural signals along the animal are much easier to perform compared with fish, tetrapods, and hexapods, all of whom have fewer limbs and less homogeneous structures. Indeed, it is known that the central nervous system of the centipede consists of a series of paired segmental ganglia which controls the body and limb motions^15^ and centipedes can walk around even when their head, including the brain, is cut off^16^. Therefore, these findings suggest that centipedes might possess decentralized control mechanisms for generating locomotion patterns.

In this study, we employed a phenomenological mathematical modelling approach based on behavioural observations to effectively propose hypotheses and extract the essence of control mechanisms underlying amphibious locomotion. Specifically, we intensively observed the transitional behaviour between walking on land and swimming in water and predicted that local behavioural changes would become apparent at the boundary of the two different environments. In addition, we conducted ventral nerve cord transection in the middle of the centipede body to examine the role of descending control from the brain. Combining these behavioural experiments, we could capture the longitudinal change in locomotor pattern during substrate transition and found that mechano-sensory feedback based on ground contact inhibits swimming directed from the brain and induces walking. We performed neuro-mechanical simulations and demonstrated that our simple model could reproduce versatile adaptive behaviours in centipedes.

## Results

### Amphibious locomotion of the centipede *Scolopendra subspinipes mutilans*

Terrestrial locomotion of a centipede was observed on a flat terrain (Fig. 1a, Supplementary Movie S1). The centipede walked by propagating the wave of leg movement posteriorly. Notably, body undulation was hardly seen during the straight walking. Aquatic locomotion of the centipede in water is shown in Fig. 1b (Supplementary Movie S2). The centipede swam with a posteriorly travelling wave of body undulation with the legs folded along the body side except for a few anterior legs that mostly remained unfolded.

**Figure 2:**
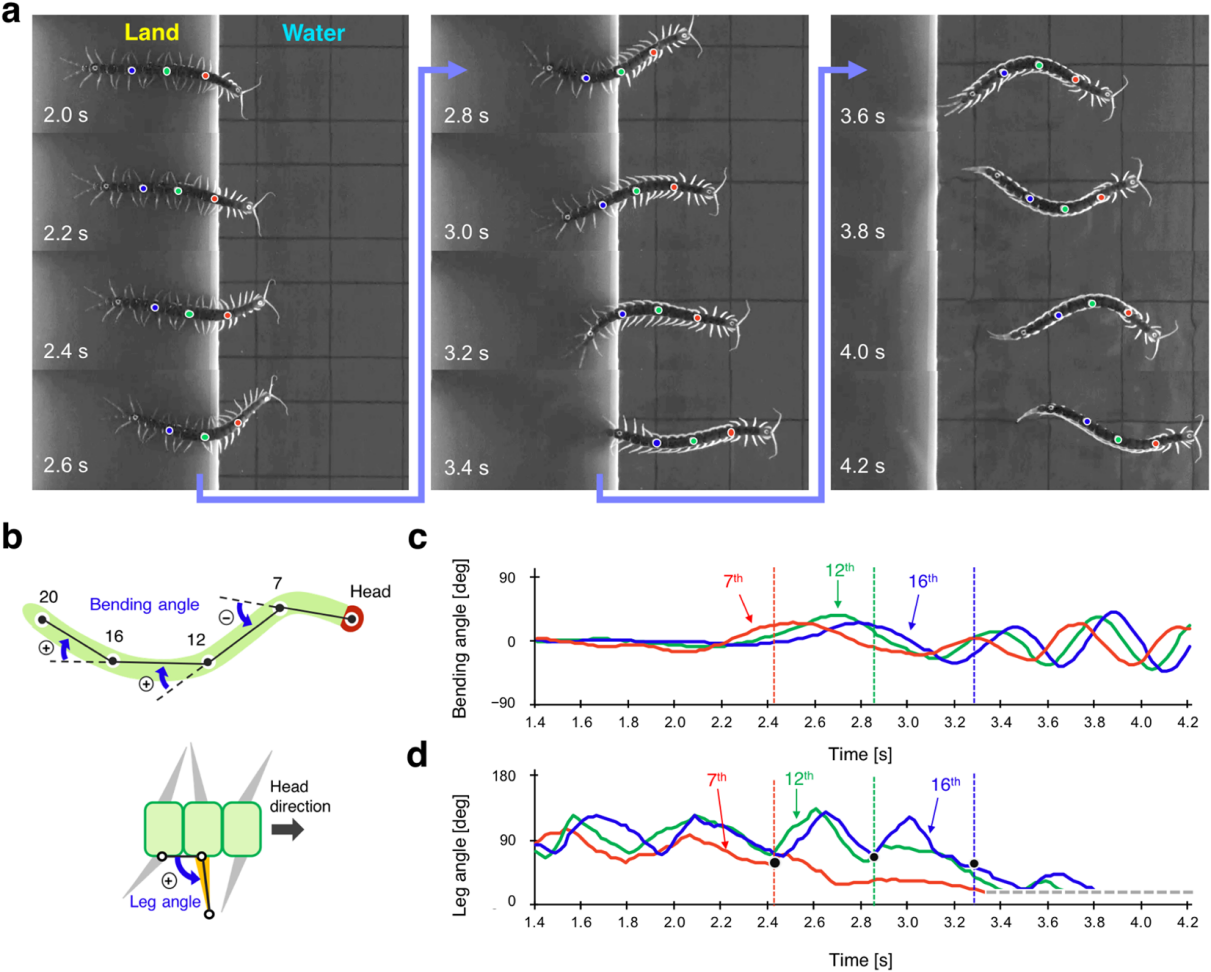
Transition from land to water. (**a**) Snapshots from the top view taken every 0.2 s. Red, green, and blue circles marked on the centipede body denote the 7th, 12th, and 16th segment, respectively. (**b**) Definition of the measured body bending angle and leg angle. (**c**) Bending angles of the body trunk. (**d**) Angles of the right legs. Red, green, and blue lines in the graphs denote the angles of the 7th, 12th, and 16th segment, respectively. Grey dashed line in the graph denotes that the leg angles were smaller than 18°. The time when each leg tip entered water is indicated by solid black dots. Note that each leg continued to maintain periodic walking motion until it entered water. Qualitatively similar behaviour was observed for N = 8.

**Figure 3:**
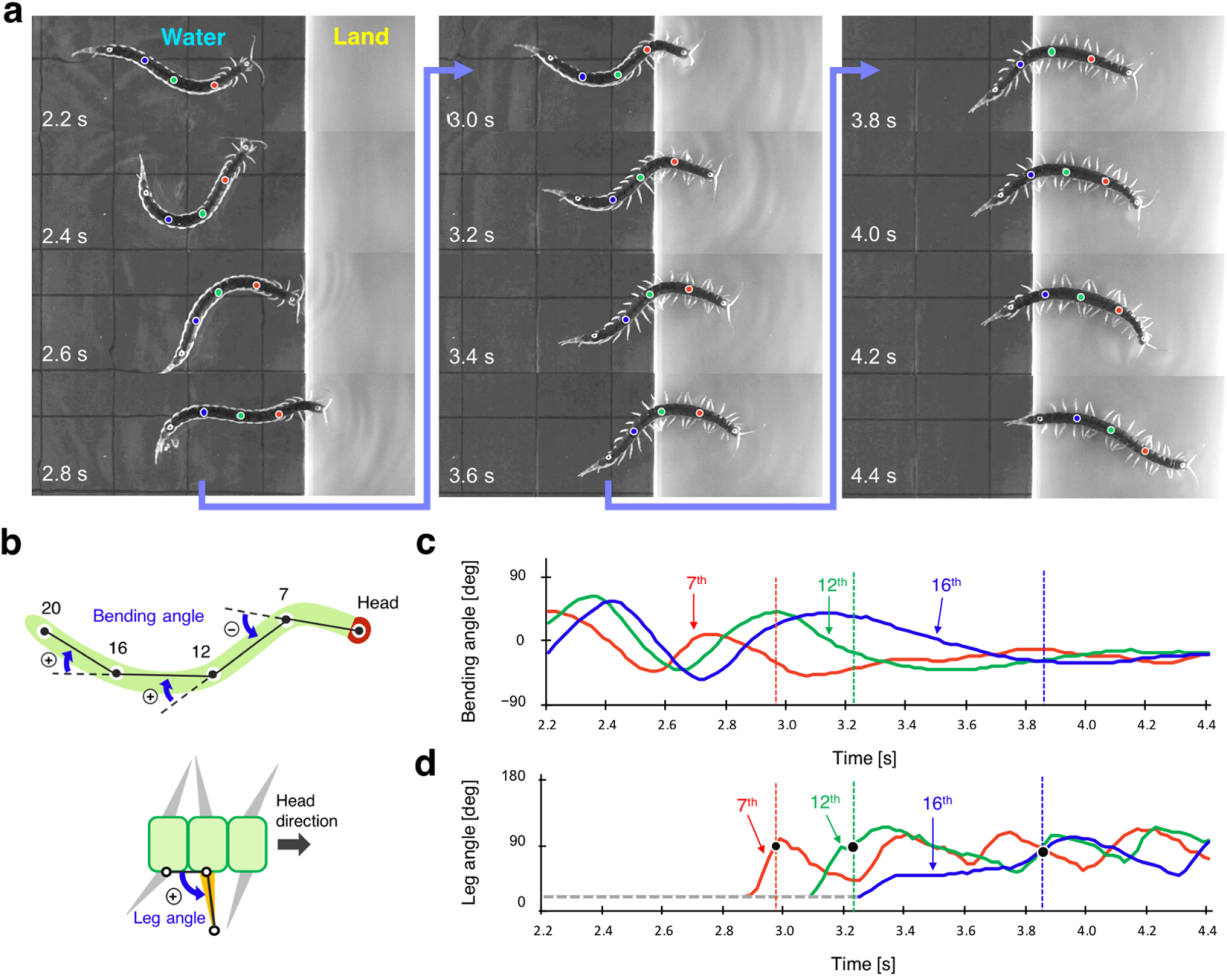
Transition from water to land. (**a**) Snapshots from the top view taken every 0.2 s. Red, green, and blue circles marked on the centipede body denote the 7th, 12th, and 16th segment, respectively. (**b**) Definition of the measured body bending angle and leg angle. (**c**) Bending angles of the body trunk. (**d**) Angles of the right legs. Red, green, and blue lines in the graphs denote the angles of the 7th, 12th, and 16th segment, respectively. Grey dashed line in the graph denotes that the leg angles were smaller than 18°. The time when each leg tip reached land is indicated by solid black dots. Note that each leg started to unfold before it reached the ground. Qualitatively similar behaviour was observed for N = 8.

To investigate the role of local sensory feedback in the switching of locomotor pattern for walking and swimming, we focused on the transitional behaviour. Representative behavioural sequences during transition from land to water and from water to land are shown in Figs. 2a and 3a, respectively (Supplementary Movies S3 and S4). In both cases, we found that the locomotor pattern gradually changed from the front part of the body. Figs. 2c,d and 3c,d show the bending angles of the body trunk around the 7th, 12th, and 16th body segment and position of the 7th, 12th, and 16th legs in the right side. As the centipede entered into the water from land (Figure 2a), each leg continued periodic walking motion, with little body bending, until the leg itself entered the water (Fig. 2c,d). Thereafter, the legs were submerged in water and began to fold along the side of the body, whereas the body bending motions became apparent gradually. Although qualitatively similar behaviour was observed for N = 8, across the trials, there was variation in the timing of initiating leg folding and body bending after each body segment entered inside water.

Conversely, when the centipede transitioned onto land from water (Fig. 3a), each leg started to unfold before it actually reached the ground and there was a delay in the start of leg oscillation for the posterior legs (Fig. 3c,d). After the legs reached the ground, they exhibited periodic walking motion, and simultaneously, the body bending motions diminished gradually. Thus, behavioural observations of centipede locomotion during the transition between land and water revealed that in response to the environmental changes, the leg and body motions of the centipede changed at the local segment level.

### Nerve cord transected centipede reveals the role of descending control from the brain

**Figure 4:**
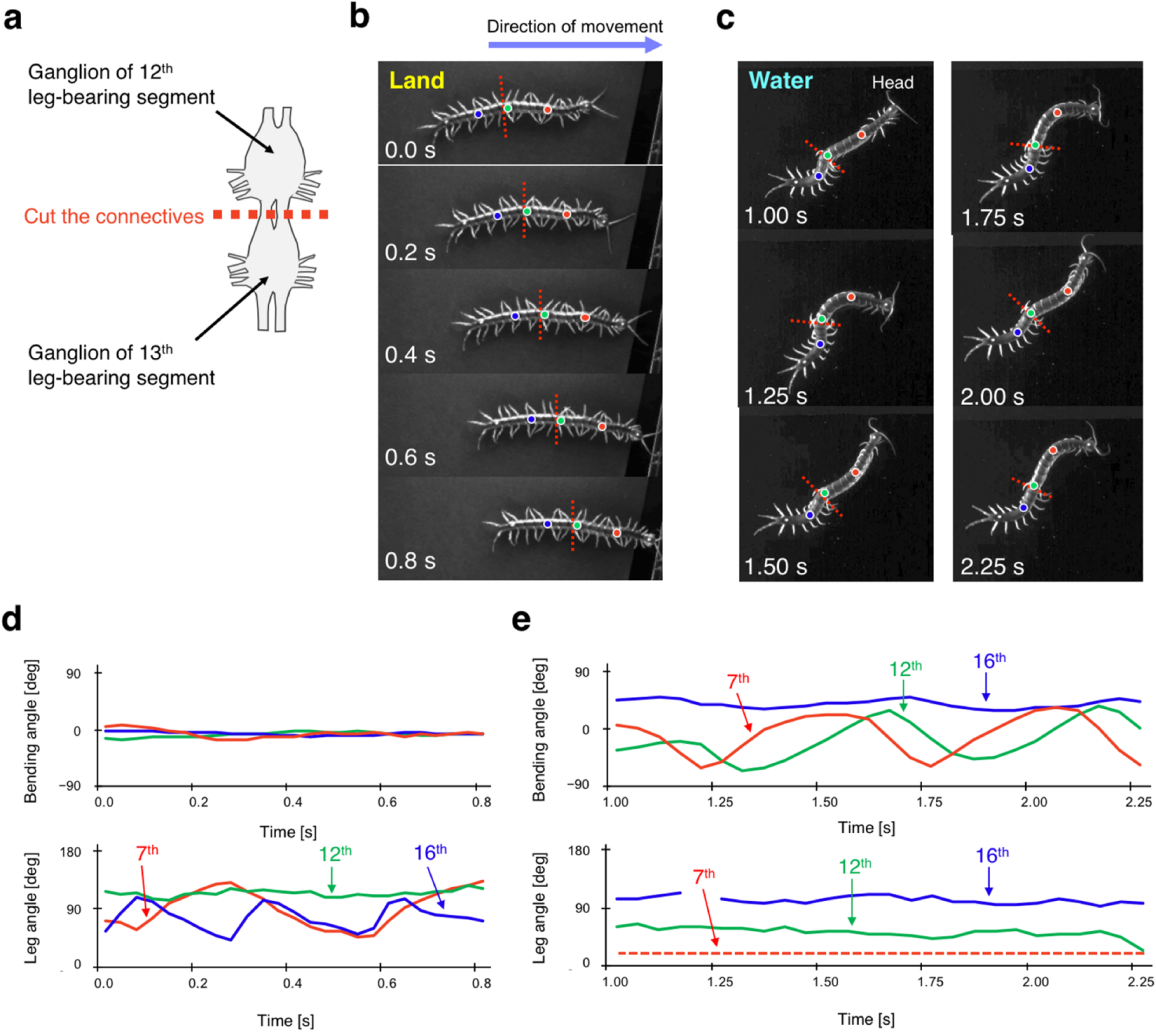
Behavioural experiments with nerve cord transected centipedes. (**a**) Schematic of nerve cord transection. Connectives of the ventral nerve cord between the ganglia in the 12th and 13th body segments were transected. (**b**) Walking pattern of a nerve cord transected centipede. (**c**) Swimming pattern of a nerve cord transected centipede. Snapshots were taken every 0.2 s (**b**) and 0.25 s (**c**). Red dotted lines denote the region of nerve transection. Red, green, and blue circles marked on the centipede body denote the 7th, 12th, and 16th segment, respectively. (**d**) Bending angles of the body trunk and angles of the right legs during locomotion on land. Notably, legs at the 12th segment did not move because the ganglion of 12th segment was damaged due to the transection surgery. (**e**) Bending angles of the body trunk and angles of the right legs during locomotion in water. Red, green, and blue lines in the graphs denote the angles of the 7th, 12th, and 16th segment, respectively, and all the angle data were measured similarly as defined in Figs. 2b and 3b. Red dashed line denotes the angles of the leg at the 7th segment were smaller than 18°. Qualitatively similar behaviour was observed for N = 7.

To investigate the effect of descending control from the brain on locomotor pattern generation, we transected the connectives of the ventral nerve cord between the ganglia in the 12th and 13th body segments (Fig. 4a, see Methods section in detail). On land, the nerve cord transected centipede immediately exhibited walking in both anterior and posterior body sections, and interestingly, the legs showed coordinated patterns similar to intact animals (Fig. 4b,d, Supplementary Movie S5). Inside water, the centipede exhibited swimming with body undulation and leg folding in the anterior section, whereas the posterior section of the body trunk showed little movement, with the legs being paused in the unfolded position, exhibiting almost no movements (Fig. 4c,e, Supplementary Movie S6).

### Mathematical model for flexible locomotor pattern switching

**Figure 5:**
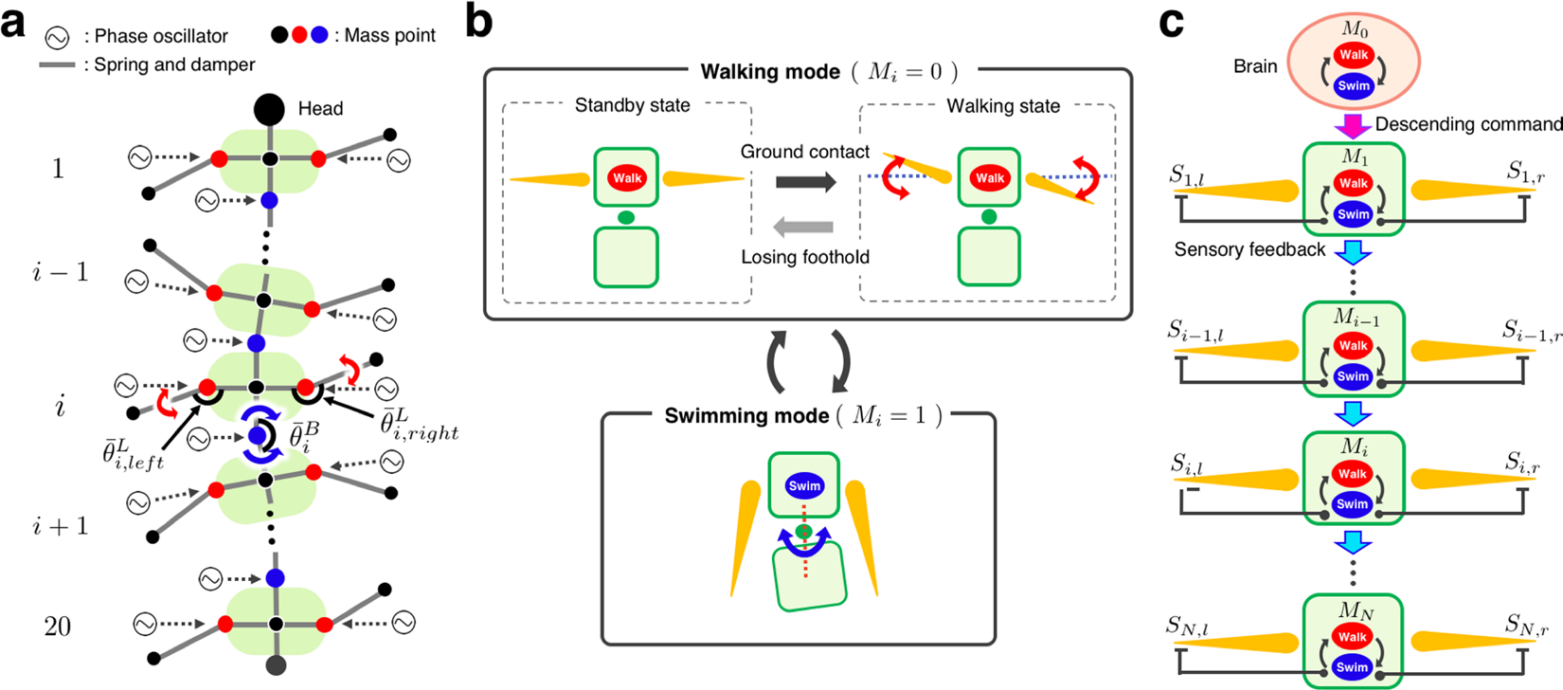
Schematic of the proposed model. (**a**) 2D physical model of a centipede. Circles denote mass points and straight lines between the circles denote parallel mechanisms of a rigid spring and a damper. A phase oscillator is implemented in each of the leg joint (red circle) and body joint (blue circle). (**b**) Hypothesized locomotor behaviour depending on the locomotor modes of walking and swimming in each body segment. (**c**) Schematic of the proposed control mechanism which determines the locomotor mode in each segment.

The aim of this study was to capture the essential interplay between the descending control from the brain, local pattern generating circuits (CPGs), and sensory feedback during amphibious locomotion of centipedes. Therefore, we constructed an integrative neuro-mechanical simulation based on a simple 2D physical model (Fig. 5a). The centipede body is described as a mass-spring-damper system, and each of the leg and body trunk has one rotational degree of freedom for leg swinging and body bending. In this study, we assumed that the periodic motions of the leg and body trunk are controlled according to the phase oscillators implemented in each actuator, which correspond to the CPGs in biological system. Although many studies have investigated the detailed mechanisms for coordination between legs (i.e. interlimb coordination)^17^, and that between parts of the body trunk (i.e. intersegmental coordination)^18^, for simplicity, our abstract model assumed that the travelling waves of the leg tip positions and body bending are generated by neural couplings between the leg and body trunk oscillators (see Methods section in detail). This simplification allowed us to concentrate on describing the mechanism responsible for switching between walking and swimming by descending control from the brain and sensory feedback.

From our abovementioned behavioural experiments, some speculation on the control mechanisms underlying centipede locomotion can be derived. The locomotion of nerve cord transected centipedes suggests that walking motion can be generated even in the absence of descending control from the brain (Fig. 4b), whereas the initiation of swimming motion (i.e. leg folding and travelling body undulation) needs a descending control from the brain (Fig. 4c). Furthermore, locomotion during the transition between land and water (Figs. 2 and 3) suggests that the mechano-sensory feedback based on the ground contact of each leg induces and maintains the walking pattern at the local segment level. Moreover, the change in individual leg positions during the transition from water to land (Fig. 3d) suggests that the switch of locomotor pattern from swimming to walking propagates from the anterior segment to the posterior segment via some neural pathway.

Our model mainly consists of the following four hypotheses. **Hypothesis 1**: Each body segment has two locomotor modes, walking and swimming (Fig. 5b). Herein, we define the locomotor modes as follows: During walking mode, the legs are unfolded and away from the body with little body bending and they move periodically if they detect ground contact, otherwise they do not move. Conversely, during swimming mode, the legs are folded along the body side and periodic bending motion of body trunk appears. **Hypothesis 2**: The brain can switch between the two locomotor modes and transmit the decision as a descending command to the first body segment (Fig. 5c). **Hypothesis 3**: Each body segment basically follows the locomotor mode of its anterior segment. **Hypothesis 4**: Mechano-sensory feedback based on ground contact can inhibit swimming mode and induce walking mode (Fig. 5c).

To describe the hypothesized control mechanism mathematically, we introduced a variable ${M}_{i}$. Herein, ${M}_{i}$ $(i\,=\,1,2,\,...,\,N)$ denotes the locomotor mode of *i*th body segment, whereas ${M}_{0}$ denotes the descending command from the brain. The variables ${M}_{i}$ $(i=0,1,2,...,N)$ take real values from 0 to 1, and when ${M}_{i}=0$, the segment behaves completely as in the walking mode, i.e., the legs are unfolded and the body trunk does not bend. When ${M}_{i}\mathrm{=1}$, the segment behaves completely as in the swimming mode, i.e., the legs are folded and the body trunk bends with large amplitude. Note that when ${M}_{i}$ takes an intermediate value (e.g. ${M}_{i}=0.5$), the neutral position of the legs shifts backward as compared to the walking mode and the body trunk bends with small amplitude, which corresponds to a transient state between walking and swimming. According to the abovementioned hypotheses, the time evolution of ${M}_{i}$ can be described with the following equation:

1 $${\tau }_{M}{\dot{M}}_{i}=-{M}_{i}+{\rm{m}}{\rm{a}}{\rm{x}}[0,{M}_{i-1}-\sum _{j\in \{left,right\}}{S}_{i,j}],i\in {\mathbb{N}},$$

where, ${\tau }_{M}$ is the time constant and ${S}_{i,j}$ $\mathrm{(0}\le {S}_{i,j}\le \mathrm{1)}$ denotes the activity level of the mechano-sensory neurons that burst when the *i*th leg in the *j* side detects a force larger than the threshold. Note that this model assumed ${\tau }_{M}$ as a constant for simplicity, though it may vary in response to the situations. Moreover, we assumed that the legs, when inside water, are subject to below-threshold forces (${S}_{i,j}\mathrm{=0}$) and ${S}_{i,j}$ becomes positive only when they receive reaction force from the ground (see Eq. (2) in the Methods section). Equation (1) means that the locomotor mode of each segment converges to the swimming mode (*M*_*i*_ = 1) only when the segment receives a signal for swimming from its anterior segment (${M}_{i-1}$ = 1) and when there is no sensory input from the legs of its own segment (${\sum }_{j}{S}_{i,j}=0$); contrarily, it will tend to exhibit walking mode (${M}_{i}=0$). For transecting the nerve cords between the *i*th and *i*$-1$th segments, we assumed that the signal for swimming mode from the brain cannot be transmitted to the *i*th segment (${M}_{i-1}=0$); thus, the body segments posterior to the transected nerve cord definitely exhibits walking mode (${M}_{i}=0$). Please see Methods section for the details of the model.

### Model well-reproduced versatile adaptive locomotion of centipedes

To validate our hypothesized control mechanism, we simulated the centipede locomotion in various situations (see Methods section for the details of the simulation set-ups). In all experiments, we set the descending command from the brain (*M*_0_) to automatically switch between walking (*M*_0_ = 0) and swimming (*M*_0_ = 1) in response to the environment (land or water) in which the head mass point of the centipede was positioned.

**Figure 6:**
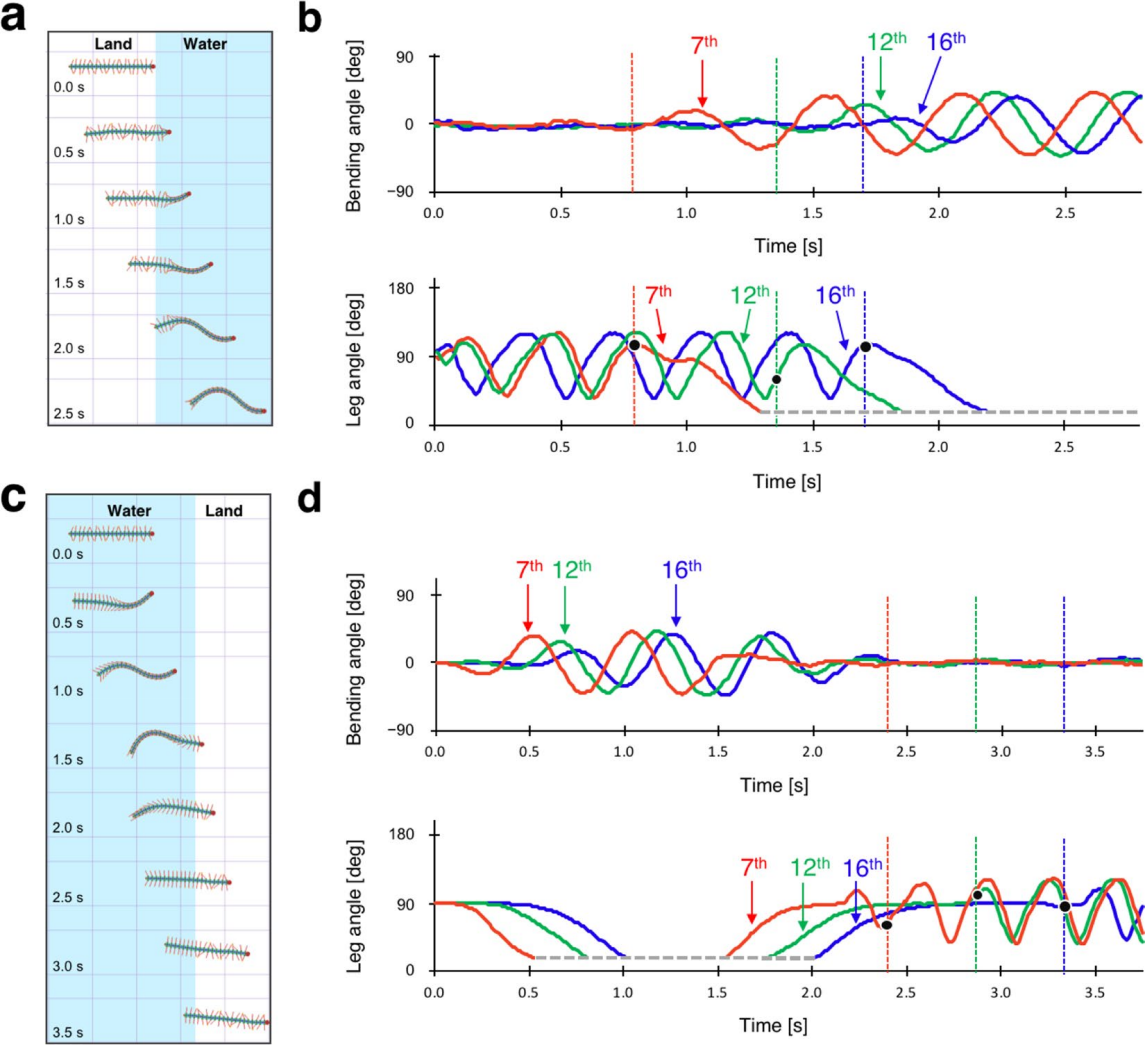
Simulation results of an intact centipede. (**a**,**c**) Snapshots of the simulated centipede during the transition from land to water (**a**), and from water to land (**c**). Snapshots were taken every 0.5 s. Blue and white areas denote water and land, respectively. (**b**,**d**) Bending angles of the body trunk (upper figure) and angles of the right legs (lower figure) corresponding to (**a**,**c**), respectively. Red, green, and blue lines in the graphs denote the angles of the 7th, 12th, and 16th segment, respectively, and all the angle data were measured similarly as defined in Figs. 2b and 3b. Grey dashed line in the graph denotes that the leg angles were smaller than 18°. The time when each leg tip entered water/land is indicated by solid black dots.

First, we simulated intact locomotion. Figure 6a shows the transition from land to water (Supplementary Movie S7). On land, the simulated centipede walked by propagating a wave of leg movement, keeping the body trunk almost straight. Thereafter, the front part of the body switched to swimming motion as the centipede lost its footholds in water, whereas the posterior part, which was still on land, continued walking (Fig. 6a,b). This result showed that the sensory feedback from the legs overrode the swimming mode directed from the brain and maintained walking mode at the posterior part which was on land. Figure 6c shows the transition from water to land (Supplementary Movie S8). In water, the simulated centipede folded its legs and started to bend the body trunk in a travelling wave form. When the front part of the body made contact with the ground, it immediately transitioned to walking, and simultaneously, the posterior legs which was still submerged in water started to unfold before they actually reached the ground area (Fig. 6d). Thus, our model could reproduce qualitatively the same walk-swim transitions in response to environmental changes by a combination of descending command from the brain and local sensory feedback.

**Figure 7:**
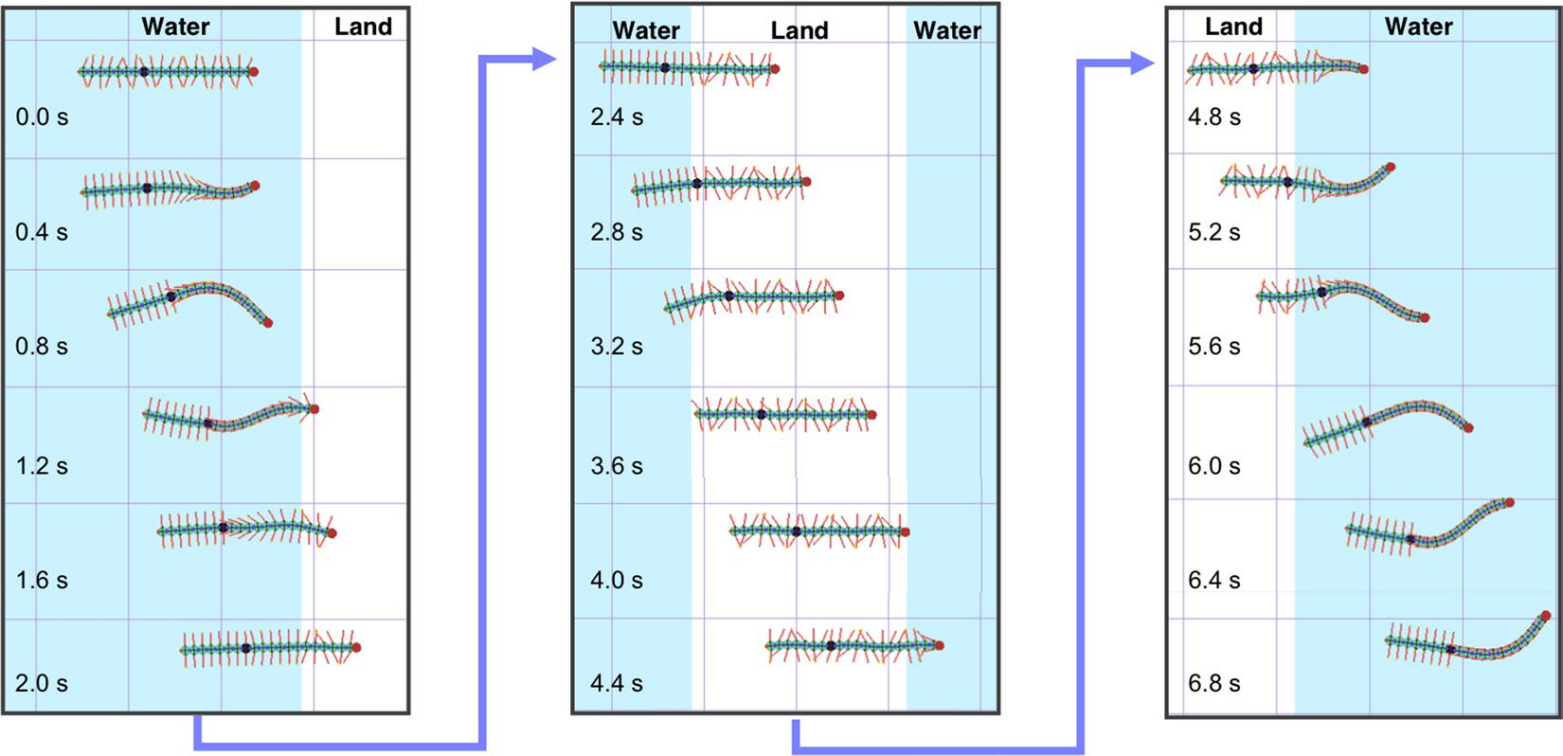
Simulation results of a nerve cord transected centipede. Neural connection between the 12th and 13th segments was transected, and the 13th segment was black coloured. Blue and white areas denote water and land, respectively. Snapshots were taken every 0.4 s.

Second, we also simulated the locomotion of a nerve cord transected centipede. The neural connection between the 12th and 13th segments was removed in our model (see Methods section in detail), and Fig. 7 shows the transitional behaviour of a simulated centipede between land and water (Supplementary Movie S9). In water, the segments anterior to the transection exhibited swimming motion, whereas the posterior segments did not move and maintained the unfolded position of the legs. In contrast, on land, the transected centipede simulation exhibited walking in all segments, even in those posterior to the transection. Thus, amphibious locomotion of the nerve cord transected centipede (Fig. 4) was reproduced successfully. Furthermore, during the simulated transition from water to land, the legs at the posterior segments initiated walking motion after they actually made contact with the ground. Conversely, during the simulated transition from land to water, the posterior legs kicked the ground until they were submerged in water, and thereafter, they did not fold inside water. Qualitatively similar behaviours to these transitions were also observed in real nerve cord transected centipedes (Supplementary Movies S10 and S11).

Third, another possible control mechanism was tested using our model framework. In this mechanism, we assumed that the brain has connections to each segment individually and can send descending signals to switch the locomotor mode simultaneously (see Eq. (12) in the Methods section). Consequently, this control structure could not reproduce the behaviour of real centipedes, especially the transition from water to land, because the whole body immediately turned to walking mode after the brain switched the descending command from swimming to walking (${M}_{0}=1\to 0$) when the head entered land (Supplementary Movie S12). This result may support our hypotheses that a neural control mechanism might exist which propagates the switch of locomotor mode from the brain to the posterior section along the entire body, through each segment, following the locomotor mode of its anterior segments (**Hypotheses 2** and **3**).

Additionally, herein we mention that our model could also reproduce the adaptive and resilient walking motion of centipedes observed in a previous study^19^. It was reported that when a part of the terrain was removed during walking on land, legs above the gap gradually stopped periodic walking motion and paused at the unfolded position, whereas the other legs in contact with land continued walking. Our model can generate this behaviour (Supplementary Movie S13) owing to the local reflexive control during walking mode: each leg pauses in the unfolded position until it detects ground contact (Fig. 5b). Moreover, we examined the locomotor behaviour when some of the legs were amputated in simulation. The simulated centipede could walk on land even when three pairs of leg in the middle segments were amputated (Supplementary Movie S14). Thus, our model also captures the fault tolerance in centipede walking.

## Discussion

The significant contribution of our model to biology is that it shows the interplay between descending control from the brain, CPGs, and sensory feedback in a simple manner. This was achieved by a synthetic approach^20^ wherein we built a highly abstract model based on behavioural experiments and tested it with simulations. Our main findings in centipedes are as follows: First, nerve cord transected centipedes revealed that walking can be initiated by mechano-sensory inputs alone, whereas swimming needs a descending control from the brain for initiation. Second, we found that the sensory feedback from the legs can override a swimming pattern directed from the brain and elicit a walking pattern. Owing to its simplicity, the essential control mechanism described in our model can be a basis for discussing common principles of locomotion control among various animal species whose detailed structures of neural and mechanical systems are different but may operate on similar mechano-sensory feedback systems. Indeed, walking function in the absence of descending control from the brain is consistent with the studies on stick insects^21^, which suggests that sensory feedback coordinates local neural oscillators (i.e. CPGs) in the legs, and also consistent with those in decerebrate cats, where gait changes are elicited by increasing mechano-sensory input from the limbs by varying the speed of treadmill^22,23^. Furthermore, the abovementioned second finding provides a new insight to the motor control in amphibious animals since it suggests that different locomotor patterns, such as walking and swimming, can be switched via local sensory feedback, as well as by the descending command from the brain, which has not been elucidated in salamanders^12,13,24^.

This study also contributes to robotics. In the field of amphibious robots^25,26^, a centipede-like robot based on our model has the following two advantages. First, it can utilize two different locomotion to achieve effective propulsion depending on the environment; legged locomotion on land and undulatory locomotion in water. Second, the robot can be fault tolerant to physical damages owing to its redundant multi-segmented body and its decentralized control. Therefore, robotic implementation of our centipede model will pave the way to realize a highly adaptive and resilient amphibious robot.

Finally, we note that our model still has some limitations. First, the model could not generate walking motion on an extremely slippery surface, whereas a real centipede can (Supplementary Movie S15). This is because our model assumes that legs exhibit walking motion only when they receive sufficient mechano-sensory inputs through their interactions with the ground. Thus, centipedes may have other control mechanisms to generate walking even in the absence of mechano-sensory inputs from the legs. Second, the model could not reproduce the adaptive centipede locomotion in response to varying locomotion speed. As is known in various animals^27,28^, the centipede *Scolopendramorpha*, changes the locomotion patterns from low-speed to high-speed walking; it exhibits body undulation combined with legged motion as the locomotion speed increases^29^. Because we assumed that walking is a locomotor mode solely exhibited by leg motions (with no body undulation) for simplicity, the present model cannot reproduce high-speed walking. However, we believe that exploring the role of descending control in changing locomotion speed with neurophysiological experiments will lead to the extension of our model and improve the understanding of motor control in centipede locomotion.

## Methods

### Behavioural experiments

All centipedes (*Scolopendra subspinipes mutilans*) were wild-caught in Wakayama, Japan, and a total of 15 individual centipedes were used for behavioural experiments. Eight centipedes were used for intact observations in terrestrial and aquatic environment, and their body length and weight were 8.7 ± 0.7 cm and 2.2 ± 0.4 g. Seven centipedes were used for ventral nerve cord transection experiments, and their body length and weight were 10.6 ± 0.9 cm, 2.9 ± 0.6 g.

All experiments were recorded by high-speed video cameras (DITECT, type HAS-U2). In the behavioural experiments with intact centipedes (Figs. 1, 2a and 3a; Supplementary Movies S1–S4), videos were recorded at a resolution of 1280 × 1024 pixels and a frame rate of 150 frames per second (fps). In the nerve cord transected experiments (Fig. 4b,c; Supplementary Movies S5, S6, S10 and S11), videos were recorded at 1024 × 768 pixels, 300 fps for walking and 200 fps for swimming.

To collect the kinematic data of centipede locomotion, five markers were painted with white nail polish and black oily ink (for intact centipedes) and white oily ink (for nerve cord transected centipedes) on the body as follows: One was on the top of the head and the other four were on the dorsal surface of the 7th, 12th, 16th, and 20th body segments. Bending angles of the body trunk and angles of the right legs (Figs. 2c,d, 3c,d and 4d,e) were measured by analyzing the video clips using the image software, Tracker.

In the experiment of intact centipedes, we used a flat terrain covered with polyethylene plate for land (Figs. 1a, 2a and 3a; Supplementary Movies S1, S3 and S4), and the water used was about 25 °C and 4.5 cm deep (Figs. 1b, 2a and 3a; Supplementary Movies S2–S4). In addition, the land area during the transition experiments (Figs. 2a and 3a; Supplementary Movies S3 and S4) was 3 mm under water to make the centipedes’ transition to different environments smooth. Note that the body height at this depth is higher than 3 mm and the leg tips make contact with the ground. In the experiments with nerve cord transected centipedes, we used a flat terrain covered with a black drawing paper for land (Fig. 4b; Supplementary Movie S5), and the water used was about 23 °C and 5.5 cm deep (Fig. 4c; Supplementary Movie S6).

The ventral nerve cord transection surgery was conducted as follows: The centipedes were anesthetized with CO_2_ and put on an ice-cold plate to prevent them from waking up. Using a scalpel, we opened the ventral sternite at the 12th body segment and cut the connectives of the ventral nerve cord between the ganglia in the 12th and 13th body segment. To prevent the content inside the body from flowing out, we attached the ventral sternite to the original position with an instant glue and then kept the specimen in a warm place for about one hour for recovery.

### Model

The centipede body is described as a two-dimensional model in a horizontal plane, and it consists of a mass-spring-damper system (Fig. 5a). The body has 20 segments and each of them has a pair of leg. Each leg consists of a parallel combination of a spring and a damper, and we set the spring constant for the leg at a large value to prevent the leg from expanding and contracting. The proximal end of each leg is connected to the body trunk with a damper and a torsional real-time tunable spring (RTS)^30^ whose natural angle can be changed arbitrarily; thus, it can work as an actuator. Mass points of the body trunk are connected in the posterior-anterior direction with a joint which consists of a damper and a torsional RTS. Accordingly, the centipede model has one rotational degree of freedom in the yaw direction for each leg and body trunk. To describe the periodic rhythms of leg and body motion, we implemented a phase oscillator in each actuator. Each leg is supposed to swing forward and backward when the leg oscillator phase (${\phi }_{i,j}^{L}$: “$i$” denotes the segment number and “$j$” denotes left or right side of the body.) is between 0 and $\pi$, and between $\pi$ and 2$\pi$, respectively. The body trunk is supposed to bend leftward to form the rightward convex shape when the body oscillator phase (${\phi }_{i}^{B}$) is between 0 and $\pi$, and rightward when the phase is between $\pi$ and 2$\pi$.

The interaction forces between the body and environment are described as viscous friction for simplicity. Each mass point receives viscous frictional force, proportional to its velocity, from the environment (the coefficients of viscous friction are summarized in Supplementary Table S1). When the body is on land, we assumed that the leg tip lifts off the ground during $0\le {\phi }_{i,j}^{L} < \pi$ and makes contact with the ground during $\pi \le {\phi }_{i,j}^{L}\mathrm{ < 2}\pi$. Thus, the friction coefficient of the leg tip mass during $0\le {\phi }_{i,j}^{L} < \pi$ is set to be zero. In addition, the friction coefficient of the body trunk masses is set to be zero since the body trunk of a real centipede mostly lifts off the ground during walking. Meanwhile, when the body is in water, the whole body always interacts with the environment, and thus, the friction coefficients have non-zero values. We assumed that the friction coefficient of the leg tip masses in water (${\mu }_{w}$) is smaller than that on the ground (${\mu }_{g}$). Moreover, it is also assumed that the friction coefficient of the body trunk masses in the tangential direction (i.e. forward-backward direction), ${\mu }_{t}$ is set to be smaller than that in the normal direction (i.e. lateral direction), ${\mu }_{n}$.

Accordingly, we have described the activity level of mechano-sensory neurons in each leg (${S}_{i,j}$) as follows:

2 $${\tau }_{S}{\dot{S}}_{i,j}=-{S}_{i,j}+{\rm{m}}{\rm{a}}{\rm{x}}\mathrm{[0,}{\rm{t}}{\rm{a}}{\rm{n}}{\rm{h}}\{{c}_{S}(|{\overrightarrow{F}}_{i,j}^{tip}|-{F}_{th})\}],$$

where, ${\tau }_{s}$ is the time constant, ${c}_{S}$ is the positive constant, $|{\overrightarrow{F}}_{i,j}^{tip}|$ is the absolute value of reaction force from the environment (i.e. viscous friction) received at the leg tip, and ${F}_{th}$ is the threshold value. We assumed that inside water, the leg receives small force under the threshold value (i.e. ${S}_{i,j}=0$) and ${S}_{i,j}$ becomes positive only when the leg receives reaction force from the ground.

We assumed that the inter-limb and inter-segmental coordination, which represent posteriorly propagating wave of leg movement and axial bending waves, respectively, are generated by coupled phase oscillators. The time evolution of the phase of oscillator implemented in each joint is described as follows:

3 $${\dot{\phi }}_{i,j}^{L}={\omega }_{L}+{\sigma }_{1}{\rm{s}}{\rm{i}}{\rm{n}}({\phi }_{i-1,j}^{L}-{\phi }_{i,j}^{L}-{\psi }_{L}^{ipsi})+{\sigma }_{2}{\rm{s}}{\rm{i}}{\rm{n}}({\phi }_{i,k}^{L}-{\phi }_{i,j}^{L}-{\psi }_{L}^{contra})$$

4 $${\dot{\phi }}_{i}^{B}={\omega }_{B}+{\sigma }_{3}\sin ({\phi }_{i-1}^{B}-{\phi }_{i}^{B}-{\psi }_{B})$$

where, ${\phi }_{i,j}^{L}$ and ${\phi }_{i}^{B}$ are the oscillator phases of the leg and body trunk, ${\omega }_{L}$ and ${\omega }_{B}$ are the intrinsic angular frequencies of the leg and body trunk, ${\sigma }_{1}$, ${\sigma }_{2}$ and ${\sigma }_{3}$ are the positive constants, and ${\psi }_{L}^{ipsi}$, ${\psi }_{L}^{contra}$ and ${\psi }_{B}$ are the desired phase differences between the ipsilaterally neighbouring legs, contralaterally neighbouring legs and body trunks, respectively.

The joint torque at each leg (${\tau }_{i,j}^{L}$) and body trunk (${\tau }_{i}^{B}$) is determined according to proportional-derivative control as follows:

5 $${\tau }_{i,j}^{L}={k}^{L}({\bar{\theta }}_{i,j}^{L}-{\theta }_{i,j}^{L})-{d}^{L}{\dot{\theta }}_{i,j}^{L},$$

6 $${\tau }_{i}^{B}={k}^{B}({\bar{\theta }}_{i}^{B}-{\theta }_{i}^{B})-{d}^{B}{\dot{\theta }}_{i}^{B}$$

where, ${\bar{\theta }}_{i,j}^{L}$ and ${\bar{\theta }}_{i}^{B}$ are the target joint angles of each leg and body trunk, and ${\theta }_{i,j}^{L}$ and ${\theta }_{i}^{B}$ are the actual angles of each leg and body trunk, respectively. ${k}^{L},{k}^{B},{d}^{L}$, and ${d}^{B}$ are the positive constants. The target joint angle of each leg (${\bar{\theta }}_{i,j}^{L}$) is defined as follows:

7 $${\bar{\theta }}_{i,j}^{L}={\theta }_{i,j}^{neutral}-{A}_{i,j}^{L}\cos {\phi }_{i,j}^{L},$$

8 $${\theta }_{i,j}^{neutral}=\pi -{c}_{0}{M}_{i},$$

where, ${\theta }_{i,j}^{neutral}$ is the neutral angle for leg oscillation, ${A}_{i,j}^{L}$ is the amplitude of leg motion, and ${c}_{0}$ is the positive constant. Equation (8) means that the neutral leg position is changed according to the locomotor mode of the segment, i.e., the leg unfolds during walking mode and folds during swimming mode (Fig. 5b). The target joint angle of each body trunk (${\bar{\theta }}_{i}^{B}$) is defined as follows:

9 $${\bar{\theta }}_{i}^{B}=\pi -{A}_{i}^{B}{\rm{c}}{\rm{o}}{\rm{s}}{\phi }_{i}^{B},$$

where, ${A}_{i}^{B}$ is the amplitude of axial bending motion.

This model describes the switching of locomotor patterns by varying the amplitudes of the leg and body motion (${A}_{i,j}^{L}$, ${A}_{i}^{B}$) as follows:

10 $${A}_{i,j}^{L}={c}_{L}({S}_{i,j}+{S}_{i-\mathrm{1,}j}),$$

11 $${A}_{i}^{B}={c}_{B}{M}_{i},$$

where, *S*_*i*,*j*_ is the mechano-sensory information detected at the *i*th leg-tip in the *j* side (Eq. (2)), and *c*_*L*_ and *c*_*B*_ are the positive constants. Equation (10) means that the amplitude of leg oscillation becomes large when the leg itself or the one anterior makes contact with the ground. This formulation is based on the observation during transition from water to land (Fig. 3d) that the posterior leg in water starts oscillation right before it contacts the ground. Equation (11) means that the amplitude of body bending becomes large during swimming mode.

### Simulation experiments

The program for simulation was written in C++ and the simulation results were visualized using OpenGL. The differential equations were solved using the fourth-order Runge-Kutta method with a time step of 1.0 × 10^−5^ s. We set the body length and weight of the simulated centipede at 10.0 cm and 2.0 g, respectively. The leg length and mass of each leg tip were set at 0.7 cm and 2.4 mg, respectively. The parameter values employed in the simulations, which were chosen by trial and error, are listed in the Supplementary Table S2. For the first body segment, ${\sigma }_{1}$, ${\sigma }_{3}$, and *S*_*i*−1,*j*_ were set to be zero. At the initial condition of all experiments, the simulated centipede had a straight body posture (${\phi }_{i}^{B}=\frac{\pi }{2}$) and the leg-tip positions were set randomly by varying the initial phases of the leg oscillators.

In the experiment with nerve cord transected centipede (Fig. 7, Supplementary Movie S9), we performed transection of the neural connection between the 12th and 13th segment as follows: *M*_*i*−1_, ${\sigma }_{1}$, ${\sigma }_{3}$, and *S*_*i*−1,*j*_ were set to zero for control of the 13th segment. In the experiment where we tested another possible control mechanism (Supplementary Movie S12), we used the following equation for the switch of locomotor modes:

12 $${\tau }_{M}{\dot{M}}_{i}=-{M}_{i}+{\rm{m}}{\rm{a}}{\rm{x}}[0,{M}_{0}-{\sum }_{j\in \{left,right\}}{S}_{i,j}],i\in {\mathbb{N}},$$

Note that each segment does not follow the locomotor mode of its anterior segment and receives descending signal from the brain (*M*_0_) directly in this experiment. In the experiment of gap crossing walk (Supplementary Movie S13), we removed a part of the ground during walking by changing the friction coefficient ${\mu }_{g}$ from positive value to zero.

## Electronic supplementary material

Supplementary information

Supplementary Movie

Supplementary Movie

Supplementary Movie

Supplementary Movie

Supplementary Movie

Supplementary Movie

Supplementary Movie

Supplementary Movie

Supplementary Movie

Supplementary Movie

Supplementary Movie

Supplementary Movie

Supplementary Movie

Supplementary Movie

Supplementary Movie

## Data Availability

The datasets generated or analysed during the current study are available from the corresponding author on reasonable request.
