# Supplementary material for: Decoding the essential interplay between central and peripheral control in adaptive locomotion of amphibious centipedes
Source: Sci Rep. 2019 Dec 2;9:18288. doi: 10.1038/s41598-019-53258-3 (PMC6889372; doi:10.1038/s41598-019-53258-3)
Supplement: Supplementary file 1 — Supplementary information [file 41598_2019_53258_MOESM1_ESM.pdf]

Supplementary Information

Decoding the essential interplay between central and peripheral control in adaptive locomotion of amphibious centipedes

Kotaro Yasui[1,2,*], Takeshi Kano[1], Emily M. Standen[3], Hitoshi Aonuma[4], Auke J. Ijspeert[5] and Akio Ishiguro[1]

[1]Research Institute of Electrical Communication, Tohoku University, 2-1-1 Katahira, Aoba-Ward, Sendai 980-8577, Japan.

[2]Japan Society for the Promotion of Science (JSPS), 5-3-1 Kojimachi, Chiyoda-Ward, Tokyo 102-0083, Japan.

[3]Department of Biology, University of Ottawa, 30 Marie Curie Private, Ottawa, Ontario K1N 6N5, Canada.

[4]Research Institute for Electronic Science, Hokkaido University, N12W7, Kita-Ward, Sapporo 060-0812, Japan.

[5]Institute of Bioengineering, École Polytechnique Fédérale de Lausanne (EPFL), Lausanne CH-1015, Switzerland.

*Correspondence to k.yasui@riec.tohoku.ac.jp

Supplementary Table S1 | Coefficients of viscous friction employed in simulations

	Leg tip		Body trunk	
	$0 \leq \phi_{i,j}^{L} < \pi$	$\pi \leq \phi_{i,j}^{L} < 2\pi$	tangential	normal
**On land**	0	μ_g	0	0
**In water**	μ_w	μ_w	μ_t	μ_n
	$0 < \mu_w < \mu_g$		$0 < \mu_t < \mu_n$	

Supplementary Table S2 | Parameter values employed in simulations

Parameter	Value	Dimension
ω_L	18.0	$[\mathrm{s}^{-1}]$
ω_B	12.0	$[\mathrm{s}^{-1}]$
σ_1	50.0	$[\mathrm{s}^{-1}]$
σ_2	10.0	$[\mathrm{s}^{-1}]$
σ_3	45.0	$[\mathrm{s}^{-1}]$
ψ_L^{ipsi}	$\pi/2.7$	
ψ_L^{contra}	π	
ψ_B	$\pi/11$	
c_0	$\pi/2$	
c_L	0.75	
c_B	$\pi/18$	
τ_M	0.055	[s]
τ_S	0.14	[s]
c_S	1.0×10^3	
F_{th}	1.0×10^1	
μ_g	1.4×10^{-1}	$[\mathrm{kgs}^{-1}]$
μ_w	7.1×10^{-5}	$[\mathrm{kgs}^{-1}]$
μ_t	7.1×10^{-5}	$[\mathrm{kgs}^{-1}]$
μ_n	2.4×10^{-3}	$[\mathrm{kgs}^{-1}]$
k^L	6.0×10^{-4}	$[\mathrm{m}^2\mathrm{s}^{-2}\mathrm{kg}]$
d^L	1.5×10^{-6}	$[\mathrm{m}^2\mathrm{s}^{-1}\mathrm{kg}]$
k^B	2.1×10^{-3}	$[\mathrm{m}^2\mathrm{s}^{-2}\mathrm{kg}]$
d^B	3.0×10^{-6}	$[\mathrm{m}^2\mathrm{s}^{-1}\mathrm{kg}]$
Spring constant of leg link	70.6	$[\mathrm{kgs}^{-2}]$
Damping coefficient of leg link	2.4×10^{-1}	$[\mathrm{kgs}^{-1}]$

【**Legends for Supplementary Movies**】

Supplementary Movie S1 | Walking of the centipede *Scolopendra subspinipes mutilans.*

Supplementary Movie S2 | Swimming of the centipede *Scolopendra subspinipes mutilans.*

Supplementary Movie S3 | Centipede locomotion during transition from land to water.

Supplementary Movie S4 | Centipede locomotion during transition from water to land.

Supplementary Movie S5 | Walking of a nerve cord transected centipede.

Supplementary Movie S6 | Swimming of a nerve cord transected centipede.

Supplementary Movie S7 | Simulated centipede locomotion during transition from land to water.

Supplementary Movie S8 | Simulated centipede locomotion during transition from water to land.

Supplementary Movie S9 | Simulated locomotion of a nerve cord transected centipede.

Supplementary Movie S10 | Locomotion of a nerve cord transected centipede during transition from water to land.

Supplementary Movie S11 | Locomotion of a nerve cord transected centipede during transition from land to water.

Supplementary Movie S12 | Simulated centipede locomotion during transition from water to land, driven by a control mechanism different from our proposed model. Locomotor mode of each segment is determined by Eq. (12).

Supplementary Movie S13 | Simulated centipede walking when a part of the terrain was removed. Grey area denotes the gap appeared between the land area (white-colored).

Supplementary Movie S14 | Simulated centipede walking when three pairs of leg in the middle segments were amputated.

Supplementary Movie S15 | Locomotion of the centipede *Scolopendra subspinipes mutilans* on a slippery upslope surface. The movie was taken from the top. The ground made of plastic plate was inclined 12° with respect to the horizontal.
